# At the Interface of Urban Exposure and Infection: A Classical Case of Leptospirosis in New York City

**DOI:** 10.7759/cureus.114250

**Published:** 2026-08-10

**Authors:** Priscila Lopez, Bruna De Oliveira Cox, Tjark Schliep

**Affiliations:** 1 Infectious Disease, Harlem Hospital Center, New York City, USA

**Keywords:** anicteric leptospirosis, complications of leptospirosis, leptospirosis complications, leptospirosis with severe clinical manifestation, severe leptospirosis

## Abstract

Leptospirosis is a zoonotic spirochetal infection transmitted through exposure to water or soil contaminated with animal urine, often associated with unsanitary living conditions and environmental exposure. Early recognition and prompt initiation of appropriate antimicrobial therapy are critical to prevent severe complications, including multiorgan failure and death. We present a case of a 43-year-old man from New York City (NYC) with alcohol use disorder who was experiencing homelessness and presented with bilateral lower extremity myalgias after sleeping outdoors on a soccer field while intoxicated several days prior. He was initially managed for sepsis of unknown origin with broad-spectrum antibiotics. Subsequent diagnostic testing confirmed leptospirosis, prompting de-escalation to targeted therapy with ceftriaxone. The patient improved clinically despite developing severe acute kidney injury and hyperbilirubinemia and was discharged to complete a course of doxycycline, with a favorable outcome.

## Introduction

Leptospirosis is a zoonotic bacterial infection caused by pathogenic spirochetes of the genus Leptospira [1,2]. Leptospirosis is a significant emerging and re-emerging infectious disease, particularly prevalent in tropical and subtropical regions. Its global incidence has increased due to urbanization, climate change, flooding, and extreme weather events. While traditionally associated with occupational and environmental exposure to contaminated water, urban outbreaks are becoming increasingly recognized worldwide [2,3].

The infection may range from asymptomatic or mild illness to severe disease characterized by jaundice, acute kidney injury, pulmonary involvement, multiorgan failure, and death [2-5]. The disease remains underdiagnosed because its presentation overlaps with many other febrile illnesses, including dengue, malaria, influenza-like illness, viral hepatitis, and bacterial sepsis [2].

Leptospirosis persists in nature through animal reservoirs, primarily rodents, as well as dogs, cattle, pigs, and other mammals [1,2]. These animals excrete leptospires in their urine, contaminating soil and water sources. Humans typically become infected through abraded skin or mucous membranes following exposure to contaminated freshwater, soil, or animal urine. Human-to-human transmission is rare [1,2].

Leptospira are thin, motile spirochetes with substantial genetic diversity, including pathogenic, intermediate, and saprophytic species [2]. Following entry through the skin or mucous membranes, leptospires disseminate hematogenously. The disease is classically described in the following two phases: an initial leptospiremic phase followed by an immune phase characterized by rising antibody levels. Tissue injury results from a combination of direct bacterial toxicity and host inflammatory responses. Commonly affected organs include the liver, kidneys, lungs, meninges, heart, and eyes. In severe disease, vascular injury, capillary leak, hemorrhage, and multiorgan dysfunction may occur [2,5].

## Case presentation

A 43-year-old man with a history of alcohol use disorder and psoriasis in remission presented with progressive bilateral lower extremity pain and difficulty ambulating. The pain began approximately three days prior to presentation as a deep, dull ache involving both legs from the hips to the feet. It progressively worsened from 3/10 to 7/10 in severity, ultimately limiting his ability to walk. The pain was exacerbated by movement and improved with rest, and he described it as distinct from post-exertional muscle soreness.

Associated symptoms included fever, chills, headache, malaise, anorexia, nausea, and vomiting, with minimal oral intake limited to water. He also reported one day of non-bloody watery diarrhea consisting of approximately 10 bowel movements, which resolved spontaneously. He denied abdominal pain, urinary symptoms, trauma, recent exercise, cough, rash, insect bites, recent travel, medication use, or sick contacts.

The patient described recent psychosocial stressors, including the death of a close friend, which led to heavy alcohol consumption of approximately 10 24-ounce beers daily during the preceding two weeks. He also reported sleeping outdoors on a soccer field for two days prior to symptom onset and was uncertain about potential mosquito exposure. He resided in a shelter and had previously been independent in ambulation. He denied tobacco, marijuana, or illicit drug use.

On presentation, vital signs were notable for a temperature of 101.1°F, heart rate of 125 beats/min, respiratory rate of 19 breaths/min, blood pressure of 111/66 mmHg, and oxygen saturation of 97% on room air. He appeared ill and diaphoretic. Physical examination revealed conjunctival injection, dry mucous membranes, and tongue fasciculations. Cardiopulmonary examination demonstrated tachycardia with regular rhythm and clear lungs. Abdominal examination was benign with hyperactive bowel sounds. Musculoskeletal examination revealed marked tenderness of the bilateral calves and thighs with limited range of motion secondary to pain. No rash was observed, and neurologic examination demonstrated that the patient was alert and oriented. Initial laboratory studies were notable for thrombocytopenia (Table 1). Given concern for sepsis of unclear source, empiric broad-spectrum antimicrobial therapy with vancomycin, cefepime, and metronidazole was initiated.

**Table 1:** Initial laboratory studies. AST: aspartate aminotransferase; ALT: alanine transaminase; ALP: alkaline phosphatase; TLC: total leukocytes count

Parameters	Values	Reference range
Hemoglobin	11.9 g/dL	12-15.5 g/dL
TLC	13.06×10⁹/L	4-11×10⁹/L
Platelet count	16,000/µL	150,000-450,000/µL
Creatinine	3.4 mg/dL	0.6-1.3 mg/dL
Potassium	2.8 mmol/L	3.5-5 mmol/L
Sodium	125 mmol/L	135-145 mmol/L
Bilirubin	6.5 (direct: 4.9) mg/dL	0.2-1.2 mg/dL
AST	56 U/L	10-40 U/L
ALT	127 U/L	7-56 U/L
ALP	114 U/L	44-147 U/L
Leptospira IgM	Positive	-

His hospital course was complicated by worsening systemic involvement, including pancytopenia requiring platelet transfusion, acute kidney injury with creatinine peaking at 4.7 mg/dL, hyperbilirubinemia with total bilirubin peaking at 29.8 mg/dL and direct bilirubin at 23.6 mg/dL, and elevated creatine kinase peaking at 3,095 U/L. He also developed persistent weakness, myalgias, and jaundice.

A multidisciplinary evaluation was undertaken. Hematology evaluated for thrombotic thrombocytopenic purpura, hemolysis, disseminated intravascular coagulation, and hematologic malignancy, recommending additional diagnostic testing and close monitoring. Infectious diseases considered sepsis secondary to community-acquired pneumonia versus leptospirosis in the context of hepatic and renal dysfunction, with low suspicion for thrombotic thrombocytopenic purpura or hemolytic uremic syndrome. Nephrology attributed the oligoanuric acute kidney injury to acute tubular necrosis.

Extensive infectious workup was largely negative; however, Leptospira DNA PCR and IgM antibodies were positive, confirming the diagnosis of leptospirosis. Antibiotic therapy was subsequently narrowed. Vancomycin was discontinued after three days following negative Methicillin-resistant Staphylococcus aureus (MRSA) testing. The patient completed seven days of cefepime and metronidazole, received three days of ceftriaxone, and was discharged on doxycycline to complete a seven-day course of targeted therapy. Despite severe systemic involvement, the patient remained hemodynamically stable throughout hospitalization and did not require ICU-level care. He was discharged with outpatient follow-up with primary care and chemical dependency services.

## Discussion

This case represents a classic clinical presentation of leptospirosis and highlights its continued relevance as an underrecognized urban zoonosis in New York City [3,4,6]. Although historically considered uncommon in temperate, high-income settings, leptospirosis is increasingly being recognized in NYC, reflecting the intersection of urban ecology, socioeconomic vulnerability, and climate-related environmental exposure.

Rats are the principal reservoir for human leptospiral infection, with transmission commonly linked to contact with rat urine or contaminated environments. In densely populated urban centers such as NYC, exposure may occur through standing water, garbage accumulation, food waste areas, and deteriorating infrastructure. The NYC Health Department reported 24 human leptospirosis cases in 2023, the highest annual number recorded in the city to date, although no official annual incidence reports could be found [3]. This trend reinforces that leptospirosis remains an important, though underrecognized, public health concern in NYC.

From a public health perspective, this case demonstrates how prompt recognition and treatment can reduce morbidity and mortality. Clinicians should consider leptospirosis in patients presenting with compatible febrile illness, particularly in the setting of acute kidney injury, hepatic dysfunction, thrombocytopenia, or pulmonary involvement. They should pursue early PCR and serologic testing. Early diagnosis is critical because leptospirosis is treatable, whereas delayed recognition may result in severe multisystem disease and increased mortality [5,7].

New York City provides a unique ecological niche for leptospiral transmission. Rats are a well-established reservoir for Leptospira species, and the city’s dense infrastructure and aging housing stock facilitate sustained human-rodent contact, particularly in socioeconomically vulnerable communities [3,6,8]. Homelessness represents an additional risk factor because exposure to contaminated outdoor environments, standing water, and inadequate sanitation may increase transmission risk [7,8]. Together, these exposures underscore that infection may occur through contact with contaminated water, soil, or surfaces in environments characterized by poor sanitation, structural disrepair, and high rodent activity.

Additionally, temporal clustering of cases during unusually warm and wet months supports the hypothesis that evolving climate patterns are reshaping leptospirosis epidemiology. These environmental and social conditions position leptospirosis as an emerging infectious disease of concern in modern urban settings. Beyond environmental prevention, improving outcomes for patients with leptospirosis also depends on timely diagnosis, coordinated multidisciplinary management, and transparent healthcare systems that facilitate efficient delivery of evidence-based care. These principles are increasingly emphasized in modern healthcare delivery models [9].

This case underscores the importance of a multidisciplinary public health approach integrating clinical awareness, environmental control, and policy-level interventions. Effective prevention requires sustained investment in rodent control, improved housing and sanitation infrastructure, and targeted community education. Importantly, leptospirosis disproportionately affects populations with limited access to healthcare and safe living conditions, highlighting the role of structural inequities in infectious disease risk [4,7,10].

## Conclusions

In summary, leptospirosis in NYC exemplifies a re-emerging urban infection driven by the convergence of ecological, climatic, and social factors. This case illustrates a classic clinical syndrome while emphasizing that evolving urban environments demand renewed attention to diseases traditionally considered uncommon in high-resource settings. Maintaining a high index of clinical suspicion and promoting timely diagnosis are essential to improving patient outcomes. A multidisciplinary approach and strengthened public health preparedness are key not only to improving patient outcomes but also to building more resilient, equitable, and sustainable urban health systems capable of responding to evolving infectious disease threats.
